# Burden of mortality from cancer among adults in Addis Ababa, Ethiopia, using verbal autopsy, 2007–2017

**DOI:** 10.3332/ecancer.2022.1428

**Published:** 2022-07-14

**Authors:** Tsion Afework, Birtukan Seid, Aderaw Anteneh, Wondimu Ayele, Seifu Hagos Gebreyesus, Bilal Shikur Endris

**Affiliations:** 1 School of Public Health, Addis Ababa University, Addis Ababa 1000, Ethiopia; 2 Pastoralist Concern, Addis Ababa 1000, Ethiopia; 3 Population Services International-Ethiopia, Addis Ababa 1000, Ethiopia

**Keywords:** verbal autopsy, surveillance, premature death, common cancers, Ethiopia

## Abstract

**Background:**

Cancer is one of the leading causes of death; worldwide, there were 10.0 million cancer deaths in 2020. In Ethiopia, 51,865 people died from the disease in the same year. We aimed to describe the burden of cancer mortality, the socio-demographic and other characteristics of deceased adults in Addis Ababa from 2007 to 2017.

**Methods:**

This study was part of the Addis Ababa Mortality Surveillance Programme. Based on the burial-based surveillance, there were 133,170 adult deaths from 2007 to 2017. The standard verbal autopsy questionnaire was applied to collect information on the causes of death of 10% of the randomly selected deaths.

**Results:**

Cancer accounted for 11% of all deaths studied. The median age of death in years was 60 (range = 47–70). Stomach cancer was the leading cause of cancer death (131, 13.6%), followed by breast cancer (116, 12.0%) and liver cancer (101, 10.5%).

**Conclusion:**

Cancer-related deaths accounted for a significant portion of all deaths. Premature deaths accounted for majority of the deaths. Cancer deaths were most commonly caused by stomach, breast and liver cancers. Advocating for a healthy lifestyle, effective cancer screening and effective alcohol-control regulations should be tailored to the country.

## Introduction

Cancer is the leading cause of death globally and a barrier to increasing life expectancy in the 21st century [1]. The World Health Organisation (WHO) estimated 9.6 million deaths from cancer [1]. According to the Global Organisation Board of Cancer Association Network (GLOBOCAN) estimation, 693,487 and 67,573 cancer deaths occurred in 2018 in Africa and Ethiopia, respectively [2].

According to the GLOBOCAN 2020 report, lung cancer remained the leading cause of death, with an estimated 1.8 million deaths (18%), followed by colorectal (9.4%), liver (8.3%), stomach (7.7%) and female breast (6.9%) cancers [3]. In Africa, the top five causes of cancer death were breast cancer (85,787 deaths, 12.1%), cervical cancer (76,745 deaths, 10.8%), liver cancer (66,944 deaths, 9%), prostate cancer (47,249 deaths, 6.6%) and lung cancer (41,171 deaths, 5.8%) [3].

Breast cancer was the most common cause of cancer death in Ethiopia, accounting for 9,061 (22.6%) deaths, followed by cervical cancer, which had a share of 5,338 (9.3%) deaths; leukaemia, which accounted for 3,182 (6.1%) deaths; non-Hodgkin’s lymphoma, which accounted for 2,514 (4.8%) deaths; and colon cancer, which accounted for 2,342 (4.5%) deaths [3].

Cancer has a financial impact due to premature death. It causes poverty-stricken people to die young [4]. According to GLOBOCAN, the global age-standardised death rate was 89.5%. On the other hand, 9.3% of Africans die from cancer before they reach the age of 75, and this proportion appears to be higher among women than it is among men (9.4% versus 9.3%) [3]. According to a WHO report, tobacco and alcohol use are some of the risk factors for cancer-related morbidity and mortality [5]. Additionally, lack of access to quality healthcare and comorbidities, such as diabetes mellitus, were mentioned as associated factors with mortality of cancer [6, 7]. Every year, more than 16,800 Ethiopians are killed by tobacco-related diseases such as lung cancer and other disorders [8].

One of the greatest challenges associated with collecting and analysing cancer data in many low- and middle-income countries is the lack of basic healthcare services, particularly in rural areas, resulting in many undiagnosed, untreated and, consequently, unrecorded cancer cases [9]. Ethiopia has a poor vital registration system and does not have a national cancer registry, except the one that is launched in the capital city, Addis Ababa [10]. Furthermore, socio-economic and behavioural factors associated with mortality are missing in Ethiopia. From 2013 to 2020, the WHO propelled a strategy for the early prevention and control of non-communicable diseases (NCDs), which aimed to reduce premature mortality from cancer by 25% by the year 2025 [11]. Ethiopia responded to the growing burden of cancer by launching a 5-year cancer control strategy on 26 October 2015 [12]. The strategy aimed to reduce the number of people who develop and die of cancer by expanding early detection, diagnosis and treatment, with the provision of chemotherapy, surgery, radiotherapy and palliative care [12]. Therefore, the availability of timely local epidemiologic data about the burden of disease regarding mortality is crucial to prioritise the interventions and policy related to cancer in Ethiopia. Moreover, knowing the magnitude of death from cancer and the causes of mortality is one of the foremost critical implications for assessing a country’s healthcare system.

The verbal autopsy (VA) method is a tool used for analysing the burial surveillance data. It is a reliable technique for assessing community diagnoses’ causes of deaths for less developed countries where most deaths happen outside health facilities with vital registration systems that are lacking or are weak. The WHO has developed a VA instrument for routine use to support compilation of national mortality statistics [13].

The present analysis aims at estimating the mortality burden attributed to cancer and describes the socio-demographic, health-seeking behaviours and other characteristics of deceased adults in the urban population of Ethiopia.

## Methods

### Study setting

This analysis is a portion of the data collected by the Addis Ababa Mortality Surveillance Programme (AAMSP) to ascertain the causes and trends of mortality. This programme is based in Addis Ababa, which is the capital city of Ethiopia with an estimated population of 4,793,699 [14]. Adult literacy in the capital city is the highest among all of the country’s cities. In Addis Ababa, burials are conducted at religious- or municipality-based cemeteries. Currently, there are about 73 burial sites, and the burial surveillance has been running for over two decades in all cemeteries, which is only limited to Addis Ababa city. On average, in a year, 14,800 deaths are expected to be buried in all the cemeteries.

### Study design and period

Using the surveillance design, the data used here were collected from January 2007 to December 2017, but because of the cessation of the fund, data were not available for the 2013–2014 year.

Burial surveillance sites were used as a sampling frame for the preparation of the selected sample of VA [15]. All funerals are to be conducted at either religious- or municipality-based cemeteries. The deaths are registered by cemetery clerks at each site. They register all deceased’s information, like name, date of funeral, sex, age, address, marital status, region of birth, ethnicity, religion and lay reported cause of death using a structured registration form developed by AAMSP [16]. The sources of information for cemetery clerks were relatives or close people to the deceased present during the burial ceremonies. A pair of VA data collectors, who are high school graduates, collected the VA data. They are allowed to visit a household after a grieving period of 2–3 months after the passing has happened.

**Figure 1.**
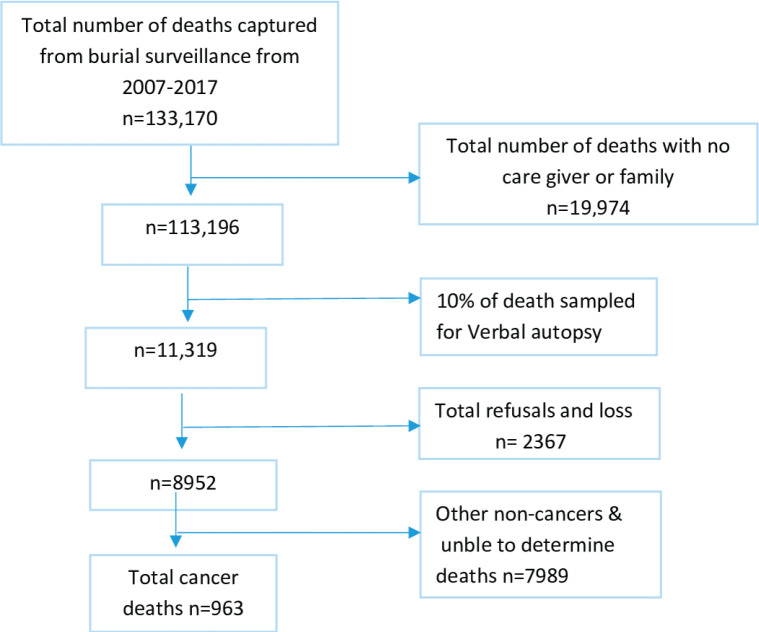
Schematic flow chart, 2007–2012 and 2015–2017, Addis Ababa, Ethiopia.

From the year, 2007 to 2017, deceased (aged 15 years and above) that were registered by the burial surveillance were 133,170; among these, 19,974 were excluded as they were without close relatives or friends who could not be potential interviewee for the VA interviews; therefore, we remained with 113,196 who were eligible for VA; thus, 10% of the deaths from the burial surveillance (11,319) were eligible for VA procedures (Figure 1).

### Verbal autopsy

VA is applied by interviewing relatives or caregivers of the deceased about the signs, symptoms, lifestyle behaviours, general circumstances surrounding the death, and other pertinent features proceeding the death [13]. These interviews were most of the time undertaken with a single close caregiver or relative who was around during the terminal illness of the deceased.

Data collectors were extensively trained on the objective, on the questionnaire and abilities of an interview with family members.

The completed VA questionnaire forms were checked by a VA research assistant in the programme office weekly. The questionnaire contains a section wherein the interviewer judges the cooperativeness and truthfulness of the responses of interviewees.

Before assigning the final underlying causes of death, all completed VAs went through physicians’ review. Primarily, two physicians reviewed the VAs independently. Any inconsistency between the two was brought up to a third physician, who either agreed with the first or second physician. If the results of the three physicians were inconsistent, then the cause of death was labelled as ‘undetermined’. In addition, there is a section where it asks if there is a medical death certificate available to show to the data collector. If there is, then the data collector will copy that and attach it with the questionnaire.

For quality checks, the VA interviews were compared with the burial surveillance record on several key variables (age, marital status, ethnicity, birth region, cause of death, etc., of the deceased) to identify possible erroneous or false interviews.

### Data management and analysis

Both the burial single and VA double data entries were implemented with the Microsoft Access, and both data sets were cleaned using STATA. For the current analysis, deaths that occurred because of cancer in the age group of 15 years and above were selected. There were 8,952 complete adult deaths with the assigned cause of death by physicians, who further analysed and focused on identifying the causes of deaths related to cancer. To extract this, we used the VA title along with the International Classification of Disease-10 code that was given by the research assistant after getting the final diagnosis assigned by the physicians.

Descriptive statistics, such as frequencies and proportions, were applied to examine the proportion of death from cancer, to see the distribution of socio-demographic, death-related information, health-seeking behaviours and lifestyle behaviours of the deceased. For continuous variables, such as age and bedridden time, we used median/Interquartile Range (IQR) after testing the assumptions of normality.

### Ethics and consent

The Institutional Review Board of Addis Ababa University, College of Health Sciences, as well as the Ethics Committee of the Ethiopian Ministry of Science and Technology had approved the programme. Permission had also been obtained from the local authorities and religious leaders for the burial sites. Written informed consent was obtained for the immediate families/caregivers of the deceased. Data were kept strictly confidential.

## Results

A total of 8,952 VA interviews were completed. Out of those, 963 (11%) died as a result of cancer.

### Socio-demographic characteristics

Table 1 shows the socio-demographic characteristics of the deceased from cancer. The median age of cancer death in years was 60 (IQR = 47–70) years and 67% died before the age of 67. More than half (586, 61%) of the deceased were female (Table 1).

### Distribution and types of adult cancer deaths

**Figure 2.**
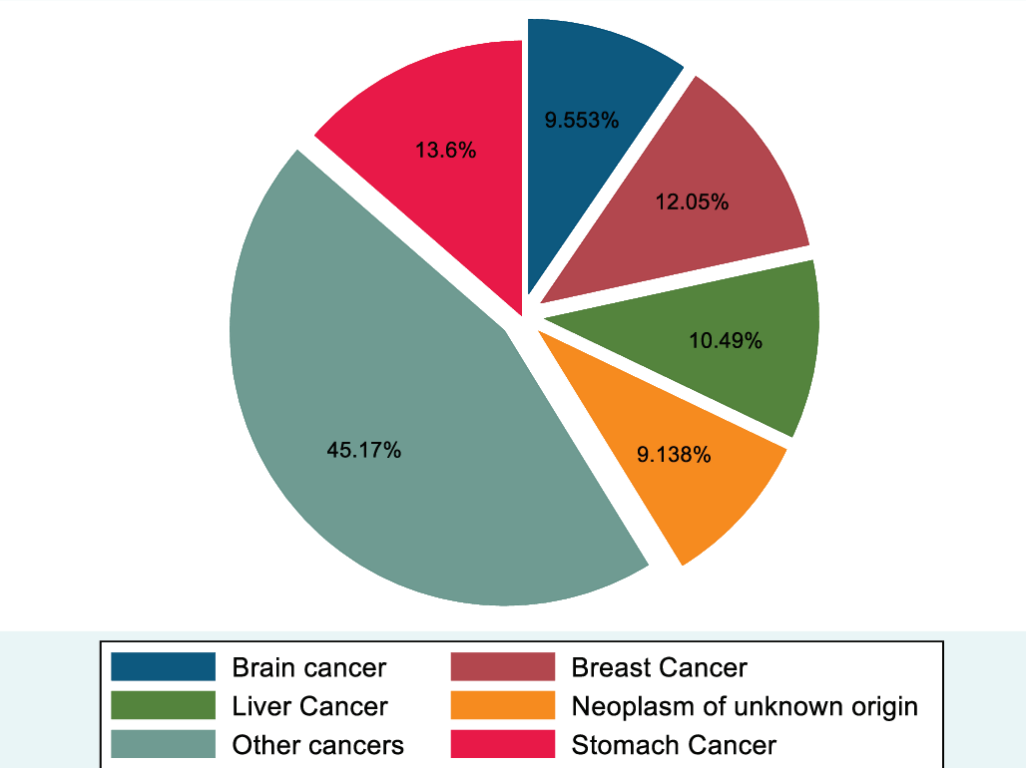
Proportion of deaths from cancer, 2007–2012 and 2015–2017, Addis Ababa, Ethiopia.

Figure 2 shows the magnitude and types of cancer deaths in both sexes in Addis Ababa. As shown, the largest share of deaths was from stomach cancer (131, 13.6%), followed by breast cancer (116, 12.0%) and liver cancer (101, 10.5%). The fourth cause of cancer death was brain cancer (92, 9.6%), and neoplasm of unknown origin ranked fifth, accounting for 88 (9.1%) deaths. All other cancers accounted for 435 (45.2%) cancer deaths (Figure 2).

### Common cancers leading to death among females

**Figure 3.**
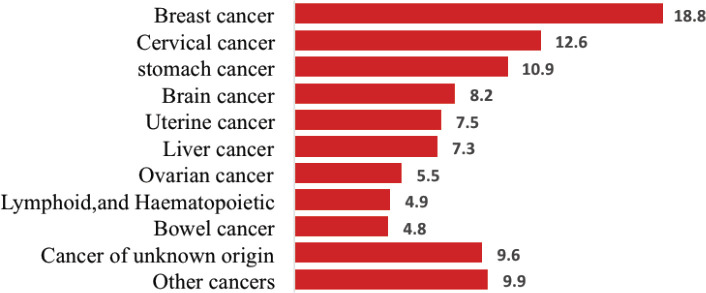
Proportion of female deaths from cancer, 2007–2012 and 2015–2017, Addis Ababa, Ethiopia.

Of the total cancer deaths, 586 (61%) were female; the leading cause of death was breast cancer (110, 18.8%), Cervical and stomach cancers accounted for 76 (12.6%) and 64 (10.9%) deaths, respectively. The top three cancers accounted for 43% of all cancer deaths among females (Figure 3).

### Common cancers leading to death among males

**Figure 4.**
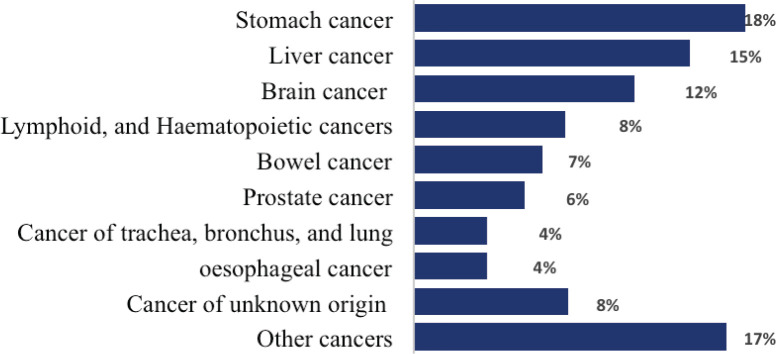
Proportion of male deaths from cancer, 2007–2012 and 2015–2017, Addis Ababa, Ethiopia.

Stomach cancer was the leading cause of death among males, which contributed to 67 (17.8%) deaths, followed by liver and brain cancers, which accounted for 58 (15.4%) and 44 (11.7%) deaths, respectively. These leading three causes of cancer death accounted for 45% of all cancer death among men (Figure 4).

### Deceased-related information

Table 2 shows that the deceased were bedridden for a median of 2 months (IQR = 0.7–5.6), and almost three-quarter of them (693, 72%) died at their home, but only 279 (29%) of them did not visit a health facility for their illness that caused death. Among the deceased, 81 (8%) were discharged from the hospitals in which they were admitted to while there were very ill (Table 2).

### Comorbidities related to cancer mortality

Table 3 shows the comorbidities that the deceased had as reported by their family members. A total of 222 (23.3%) had at least one comorbidity. The most common comorbidities reported were hypertension (108, 11%), diabetes (61, 6%) and mental illness (50, 5%) (Table 3).

### Behavioural characteristics (lifestyle of the deceased)

As reported by the family members, 272 (28%) deceased had a history of alcohol consumption. Less than 5% of the deceased used other substances, such as Khat, a flowering plant native to Ethiopia that contains the stimulant alkaloid cathinone, and smoked tobacco (Table 4).

### Association between substance use and mortality from cancer

A chi-squared test showed the association between alcohol consumption, tobacco use and Khat chewing with the top three cancer causes of death. The proportion of the deceased who had a history of tobacco use and Khat chewing did not differ by cancer type (tobacco smoke: *χ*^2^ = 6.3333, *p* = 0.176, and Khat chewing:* χ*^2^ = 3.8269, *p* = 0.430). But the proportion of deceased who had a history of alcohol consumption was different by cancer type (*χ*^2^ = 16.5225, *p* = 0.002) (Table 5).

## Discussion

This study aims to identify the burden of cancer mortality and describe the socio-demography and other characteristics of the deceased among the adult population of Addis Ababa. We used VA data from the AAMSP, from the year 2007 to 2017.

We found that cancer deaths accounted for 11% of the adult deaths. According to a WHO estimate from 2016, cancer was responsible for 7% of all deaths in Ethiopia [17]. This figure was lower in proportion when compared to our findings. This may be due to differences in the study population or data sources. On the other hand, a study conducted in China discovered an extremely high incidence of cancer deaths, with cancer accounting for 35% of all deaths [18]. According to the authors, the greater cancer mortality in China’s study could be attributable to suspected water contamination.

A study carried out in South Africa also identified that 10.2% of the deaths were due to cancer [19]. The common cancers causing death in our study were not HIV-associated cancers. Overall, there were low HIV cases reported. On the contrary, a finding in Mozambique showed that the predominant cancers are HPV-associated cancers of the uterine cervix and other AIDS-related cancers (Kaposi’s sarcoma, cancer of the conjunctiva and non-Hodgkin’s lymphomas), mirroring the increase in the HIV–AIDS epidemic in the country [20].

We found that two-thirds (67%) of the cancer deaths occurred before the age of 67, indicating a considerable number of premature deaths from cancer. Supporting this, the VA projection in India showed that 71% of all cancer deaths occurred in individuals aged 30–69 years [21]. This was similar to our finding as those who died from cancer in this age group were 66%. In African regions, 75% of premature deaths occur due to cancer [22]. In a study conducted by Misganaw *et al* [23], 58% of the deceased were in the age group of 25–64, and they concluded that NCDs contribute to premature death starting at the age of 35. Since there are differences in the cut-off point value of age classification, the proportions of premature deaths varied between the studies. However, all the studies found that cancer causes premature death [24].

We found that a higher number of women die from cancer compared to men. This could be because women outnumber men in Addis Ababa’s overall population. We are not able to further discuss this finding as we do not have the total number of male and female deaths from any cause. But other studies reported different findings as there is disproportionately higher cancer deaths among males [25]. Overall, men with any sort of cancer were 6% more likely than women to die from the condition. When men and women with the same cancer type were compared, this increased to more than 12%. This might be due to the variations between the sexes, being attributable in part to carcinogenic exposures and lifestyle factors, such as cigarette smoking, drinking alcohol and eating fattier meals, all of which are more prevalent among males [25].

On the other hand, the causes of deaths from different types of cancers were different by gender. Worldwide among men, lung cancer is the leading cause of cancer death, followed by colorectal, liver and stomach cancers [3]. The Global Burden of Diseases showed oesophageal or stomach cancer ranked first according to the number of deaths in some African countries among men [26]. Also, in 2017, stomach cancer ranked in the top five cancer causes of death for both sexes in all sub-Saharan African regions [26]. This was similar to our finding as stomach cancer ranked first in men and third in women. This similarity might be due to similar genetic predispositions and/or diet in the African regions. Liver cancer, a highly fatal cancer, appears to be the second most common cause of death for men in our study. Liver cancer was the fourth and second leading cause of cancer death in males worldwide and Africa, in 2018, respectively; it is substantially more common in developing nations [2]. This might be due to chronic viral hepatitis affecting over 70 million Africans and it is one of the main risk factors for liver cancer in Africa [27].

In India, among women, 51.2% of cancer deaths were due to cervical, stomach and breast cancers [21]. Similarly, in our study, the leading causes of death were breast cancer and cervical cancer, followed by stomach cancer, which accounted for 43%. According to GLOBOCAN, worldwide, female breast cancer is the leading cause of cancer death in over 100 countries, followed by lung, colorectal cancer and cervical cancer, which ranks fourth [2]. However, breast cancer is not the leading cause of death in many sub‐Saharan Africa countries because of the elevated cervical cancer deaths [26]. Similar to the GLOBCAN report, we found that breast cancer was the top cause of cancer death among women, which might be due to the higher incidence of breast cancer cases in Addis Ababa [10], which, in turn, can be explained by the low breastfeeding practice, low fertility rate and high obesity, which all may add up to be the risk factors for higher breast cancer incidence and mortality in Addis Ababa.

The current study provides community-based evidence about the burden and type of cancer-related deaths in Addis Ababa. The findings will help to compare similar settings and other urban areas within the country. In addition, this study provides evidence for researchers, health planners and stakeholders with limited information about the burden of cancer death. This study’s findings have significant implications for public health interventions. It can provide input to health planners and policymakers to reduce cancer-related fatalities by giving baseline information for the 5-year cancer control strategy and health system information plan.

This study should be viewed in the context of its limitations. Our findings may not apply to the deceased who were omitted from the sampling frame because they lacked close relatives or friends and had inadequate information, such as addresses. Selection biases could have existed as non-residents’ funerals might be in Addis Ababa and similarly, residents can have their funeral out of Addis Ababa. Furthermore, social desirability bias might have been introduced regarding the deceased’s behaviours/unhealthy lifestyle choices, which may have underestimated these practices. We also need to acknowledge the effects of misclassification of causes of death by the VA method. Physician review of VA questionnaires is less accurate than automated methods in determining both individual and population causes of death.

## Conclusion

As a conclusion, we found that cancer was responsible for a considerable number of deaths, resulting in premature death. The top three cancer causes of adult deaths in both sexes were stomach, breast and liver cancers. Hypertension was the most common comorbidity among the deceased, and alcohol consumption was associated with cancer-related mortality. Accordingly, cancer education, healthy lifestyle advocacy, effective cancer screening, vaccine programmes and strong alcohol-control regulations should be tailored to the country. To reduce cancer-related premature death, stakeholders should pursue comprehensive measures to prevent, control and commence early treatment. More research is needed to establish the causes of cancer-related early mortality, as well as guidelines for the prevention and treatment of female cancers should be developed and/or reinforced.

## Conflicts of interest

None of the authors have any competing interests.

## Funding

None.

## Data availability

The data sets generated during and/or analysed during the current study are available from the corresponding author on reasonable request.

## Authors’ contributions

**TA:** Conceptualisation; investigation; supervision; methodology; original draft preparation; formal analysis.

**BS:** Investigation; supervision; writing – review and editing.

**AA:** Investigation; writing – review and editing.

**WA and SHG:** Visualisation; writing – review and editing.

**BSE:** Project administration; investigation; supervision; writing – review and editing.

## Figures and Tables

**Table 1.** Socio-demographic characteristics of the deceased from cancer, 2007–2012 and 2015–2017, Addis Ababa, Ethiopia.

Variable	Frequency (*n* = 963)	Percent (CI)
**Sex** Male Female	377 586	**39.2** **60.9**
**Age** 15–19 20–24 25–29 30–34 35–39 40–44 45–49 50–54 55–59 60–64 65 ^+^	9 19 23 37 48 63 75 99 101 110 379	**0.9** **2.0** **2.4** **3.8** **5.0** **6.5** **7.8** **10.3** **10.5** **11.4** **39.4**
**Formal education** Yes No Unknown	355 603 5	**36.9** **62.6** **0.5**
**Occupation ** Housewife Manual work Professional work Sales and service Unemployed Pension Student Unknown	367 198 74 73 64 143 11 33	**38.1** **20.6** **7.7** **7.6** **6.7** **14.9** **1.1** **3.4**

**Table 2.** Deceased related information, 2007–2012 and 2015–2017, Addis Ababa, Ethiopia.

Variable	Frequency (*n* = 963)	Percent (CI)
**Duration of bedridden (by month)**	Median (IQR) 2 months (IQR = 0.7–5.6)	
**Place of death ** Home Healthcare facility On the way to the healthcare facility On holly water place Unknown	693 253 3 9 5	72 26.3 0.3 0.9 0.5
**Healthcare facility visit for the illness that caused the death**		
No	279	29.0
Yes	678	70.4
Unknown	6	0.6
**Deceased given medication during the visit**		
No	39	4.1
Yes	638	66.3
Unknown	286	29.7
**Discharged while very sick**		
No Yes Unknown	856 81 26	88.9 8.4 2.7
**Went to a traditional healer**		
No Yes Unknown	866 86 11	89.9 9.0 1.1
**Took traditional medication (*n* = 86)** No Yes	5 81	5.8 94.2
**Time of traditional medication taken compared to modern medical facility visit (*n* = 86)**		
Before	36	41.9
After	17	19.8
In between Unknown	28 5	32.7 5.8
**Visit holly water**		
No Yes Unknown	544 416 3	56.5 3.2 0.3
**Time of holly water visit compared to modern medical facility visit (*n* = 416)**		
Before	129	31.0
After	61	14.7
In between	219	52.6
Didn’t go to a hospital Unknown	5 2	1.2 0.5

**Table 3.** Comorbidities among the deceased from cancer, 2007–2012 and 2015–2017, Addis Ababa, Ethiopia.

Variable	Frequency (*n* = 963)	Percent (CI)
**Hypertension ** No Yes Unknown	844 108 11	87.6 11.2 1.1
**Diabetes ** No Yes Unknown	892 61 10	92.6 6.3 1.0
**Epilepsy ** No Yes Unknown	949 8 6	98.6 0.8 0.6
**Mental problem** No Yes Unknown	905 50 8	94.9 5.2 0.8
**HIV status** Positive Negative Unknown	8 400 555	0.8 41.5 57.6

**Table 4.** Behavioural characteristics of deceased from cancer, 2007–2012 and 2015–2017, Addis Ababa, Ethiopia.

Variable	Frequency	Percent (CI)
**Alcohol consumption ** No Yes Unknown	685 272 6	71.1 28.3 0.6
**How often (alcohol consumption) (*n* = 272)** Every day Many days in a week Every week Once in a while Unknown	42 13 6 196 15	15.4 4.8 2.2 72.1 5.5
**Khat chewing ** No Yes Unknown	915 40 7	94.9 4.2 0.7
**Frequency of Khat chewing (*n* = 40)** Every day Every week Once in a while Unknown	12 4 21 3	30.0 10.0 52.5 7.
**Tobacco smoking** No Yes Unknown	914 42 7	94.9 4.4 0.7
**Frequency of smoking (*n* = 42)** Non-stop Every hour Every day Once in a while Unknown	12 6 19 2 3	28.6 14.3 47.2 4.8 7.1

**Table 5.** Behavioural risk factors of the deceased from cancer, 2007–2012 and 2015–2017, Addis Ababa, Ethiopia.

Substances	Breast cancer		Liver cancer		Stomach cancer	
	**Frequency**	**Percent**	**Frequency**	**Percent**	**Frequency**	**Percent**
**Alcohol ** Yes No Unknown	***n* = 115** 19 94 2	16.5 81.7 1.7	***n* = 101** 40 60 1	39.6 59.4 1.0	***n* = 131** 35 96 -	26.7 73.3 -
**Khat ** Yes No Unknown	***n* = 114** 3 110 1	2.6 96.5 0.9	***n* = 100** 1 98 1	1.0 98.0 1.0	***n* = 131** 6 125 -	4.6 95.4 -
**Tobacco ** Yes No Unknown	***n* = 115** 1 113 1	0.9 98.3 0.9	***n* = 100** 7 93 -	7.0 93.0 -	***n* = 131** 5 125 1	3.8 95.4 0.8
